# A meta-analysis of prospective studies on the role of physical activity and the prevention of Alzheimer’s disease in older adults

**DOI:** 10.1186/s12877-015-0007-2

**Published:** 2015-02-11

**Authors:** Michael W Beckett, Christopher I Ardern, Michael A Rotondi

**Affiliations:** School of Kinesiology and Health Science, York University, Room 364 Strong College, 4700 Keele Street, Toronto, Ontario M3J 1P3 Canada; School of Kinesiology and Health Science, York University, Room 344 Norman Bethune College, 4700 Keele Street, Toronto, Ontario M3J 1P3 Canada; School of Kinesiology and Health Science, York University, Room 364 Norman Bethune College, 4700 Keele Street, Toronto, Ontario M3J 1P3 Canada

**Keywords:** Physical activity, Alzheimer’s disease, Older adults, Systematic review

## Abstract

**Background:**

The incidence of Alzheimer’s disease is increasing as the global population ages. Given the limited success of pharmaceuticals in preventing this disease, a greater emphasis on non-pharmaceutical approaches is needed. The aim of this study was to quantify the association between Alzheimer’s disease and physical activity in older adults over the age of 65 years.

**Methods:**

A meta-analytic approach was used to determine if physical activity reduced the risk of Alzheimer’s disease in individuals 65 years or older. Some evidence indicates that physical activity may improve cognitive function in older adults, while other evidence is inconclusive. The purpose of this study was to examine if prevention of Alzheimer’s disease is possible if started at a later age. The precise brain changes that occur with the onset of Alzheimer’s disease are not fully known, and therefore may still be influenced by preventative measures even in advancing age. Determining if physical activity can inhibit the onset of the disease at any age may motivate individuals to adopt an “it’s never too late” mentality on preventing the onset of this debilitating disease. Longitudinal studies of participants who were 65 years or older at baseline were included. A total of 20,326 participants from nine studies were included in this analysis.

**Results:**

The fixed effects risk ratio is estimated as 0.61 (95% CI 0.52-0.73) corresponding to a statistically significant overall reduction in risk of Alzheimer’s disease in physically active older adults compared to their non-active counterparts.

**Conclusion:**

Physical activity was associated with a reduced risk of Alzheimer’s disease in adults over the age of 65 years. Given the limited treatment options, greater emphasis should be paid to primary prevention through physical activity amongst individuals at high-risk of Alzheimer’s disease, such as those with strong genetic and family history.

**Electronic supplementary material:**

The online version of this article (doi:10.1186/s12877-015-0007-2) contains supplementary material, which is available to authorized users.

## Background

The world’s population is getting older, as improvements in health care and health technologies allow people to live healthier and longer lives. This increase in longevity has also contributed to an increase in the global prevalence of age-related diseases. One disease associated with aging is dementia, which is one of the major causes of disability in later life [1]. In 2011 the number of people globally living with dementia was 35.6 million, and this number is expected to double every 20 years, reaching 115.4 million by 2050 [1]. The combined financial cost for dealing with dementia on a global level was US$ 604 billion in 2010 [1]. Alzheimer’s disease is a subset of dementia and makes up the largest proportion of dementia cases [2]. Since dementia can be caused by other factors, including depression and vascular disease, this manuscript focuses exclusively on Alzheimer’s disease [3]. In 2006 there were 26.6 million cases of Alzheimer’s disease worldwide [4]. If current trends continue, it is estimated that by 2050, 1 in 85 adults will be living with Alzheimer’s and one new case of Alzheimer’s disease is expected to develop every 33 seconds [2,4].

Alzheimer’s disease causes an irreversible degeneration of brain cells that inhibits thinking ability, leading to loss of personal identity and changes in behaviours, mood and the ability to perform basic daily living activities [2,5]. Pharmaceuticals have had limited success at preventing or treating Alzheimer’s disease [5] thus a stronger emphasis on exploring a non pharmaceutical approach to preventing the onset of this disease is warranted.

The etiology of Alzheimer’s disease is multifactorial. Non-modifiable risk factors for Alzheimer’s disease include aging, family history of the disease, severe head trauma and presence of the apolipoprotein APOE4 [2,6-8]. Increasing evidence suggests that physical inactivity is also a potential risk factor for cognitive impairment and dementia in older adults [5]. While the exact mechanism of the protective status of physical activity on Alzheimer’s disease is unclear, evidence suggests that increased oxygen delivery to the brain as a result of physical activity can inhibit cell loss in the hippocampus, increase cerebral metabolic demands and preserve grey matter volume in the brain, all of which reduce the risk of cognitive impairment [4].

In the absence of a definitive understanding of this relationship we conducted a meta-analysis to determine if physical activity protects against the onset of Alzheimer’s disease in adults over the age of 65. Only adults over the age of 65 were included, as the likelihood of developing Alzheimer’s disease doubles every 5 years after this age [9]. As the population of older adults increases, a stronger emphasis should be placed on interventions targeting this specific population.

## Methods

**Figure 1:**
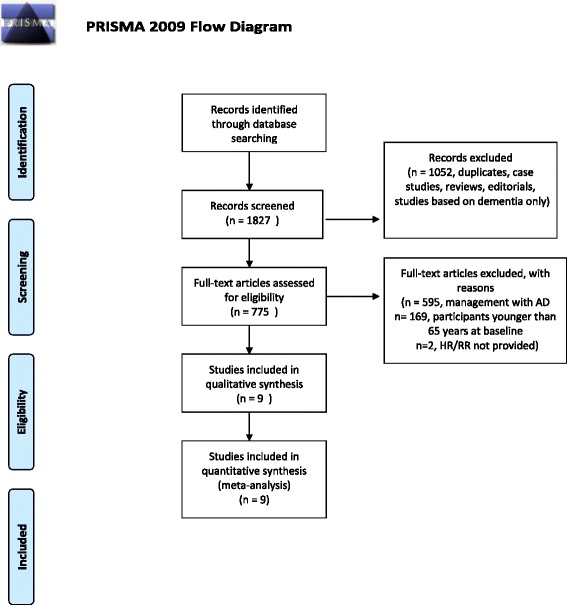
**Caption: PRISMA flowchart of review search.**

A systematic literature search of Pubmed and Google Scholar (1966 to present) was performed for prospective longitudinal studies. Longitudinal studies were included as the best available evidence for the evaluation of physical activity in the prevention of Alzheimer’s disease as long-term randomized trials are not available. Given the neurodegenerative nature of Alzheimer’s disease, which can further augment recall bias, both case control and cross-sectional studies were excluded as well. Search terms included “Alzheimer’s” and “Physical Activity”, which retrieved 1827 articles in English. Articles that clearly did not meet initial criteria were rejected on initial review. Studies were restricted to participants who were 65 years or older. All studies recorded the physical activity of participants at the beginning of the study. Studies that reported the incidence of Alzheimer’s disease as either a risk ratio (RR) or hazard ratio (HR) were included in this meta-analysis. All other outcomes were excluded. Two reviewers were responsible for reviewing and selecting the final articles. This study was guided by the PRISMA principles for the meta-analysis of observational studies in epidemiology [10] (Figure 1; Additional file 1: PRISMA Checklist) (Figure 1). As this study is based on the systematic review of previously published literature and there is no potential for participant identification, ethics approval was not required from our host institution.

Before combining studies in the meta-analysis, we evaluated the presence and possible heterogeneity in risk of Alzheimer’s disease. Confounding factors usually included age, sex, years of higher education, presence of APOE4 genotype, body mass index (BMI: kg/m^2^) and depression. Only hazard rates and risk ratios for fully adjusted models were included in this meta-analysis. All study participants had to be dementia free at study baseline. Alzheimer’s disease was diagnosed according to standardized clinical criteria (e.g., National Institute of Neurological and Communicative Diseases and Stroke-Alzheimer’s Disease and Related Disorders Association, the revised third edition of the Diagnostic and Statistical Manual of Mental Disorders and DSM-IV diagnostic criteria).

A total of ten studies met the criteria for inclusion. In one case the retrieved articles came from the same study population and were not derived from completely independent samples (Laurin et al. [11], Lindsay et al. [12]). Thus, to avoid inclusion of duplicate cohorts, Lindsay et al. [12] was included as the primary focus of the manuscript was on Alzheimer’s disease alone, rather than dementia more broadly.

A total of 1,358 participants out of 20,326 in all studies were diagnosed with Alzheimer’s disease. Descriptive characteristics for each of the included studies are presented in Table 1.

**Table 1:** **Characteristics of included studies**

**Study**	**Sample size (n)**	**Mean Length of Follow-up (years)**	**Number of Alzheimer’s cases**	**Log HR/RR**	**Log 95% C.I.**	**Mean age of study participants mean (SD)**	**%APOE (% tested positive for the APOE gene)**	**Gender (F)**	**Measurement of physical activity**
Scarmeas et al. [13]	1880	5.4	282	−0.46	(−0.82, −0.11)	77.2 (6.6)	24% n = 443	69% n = 1293	Godin-time leisure questionnaire
Buchman et al. [8]	716	4	71	−0.63	(−1.22, −0.05)	81.6 (7.12)	n/a	76% n = 602	Actigraphs worn by participants
Scarmeas et al. [14]	4166	5.2	262	−0.73	(−1.27, −0.20)	Alzheimer’s disease patients 78.8 (6.7)	Alzheimer’s disease patients, much PA: 33% n = 33 No PA: 33% n = 40	Alzheimer’s disease patients, Much Physical activity: 66% n = 71, No Physical activity: 71% n = 102	Godin-time leisure questionnaire
Larson et al. [15]	1740	6.2	107	−0.37	(−0.79, 0.05)	Participants who exercised fewer than 3x week: 74.5 (5.8) Participants who exercised more than 3x week: 74.3 (5.7)	Participants who exercised fewer than 3x week: 23% n = 100 Participants who exercised more than 3x week: 22% n = 290	Participants who exercised fewer than 3x week: 63% n = 278 Participants who exercised more than 3x week: 60% n = 772	Self-reported, regular exercise defined as “three times a week or more”
Lindsay et al. [12]	4615	5.0	194	−0.37	(−0.70, −0.04)	Alzheimer’s disease cases, 81 (sd n/a)	40% n = 39/98 cases tested	68% n = 131	Participation in regular exercise “yes/no”
Podewils et al. [7]	3375	5.4	245	−0.36	(−0.83, 0.11)	74.8 (4.9)	24% n = 813	59% n = 1,995	Minnesota Leisure time activity questionnaire
Abbott et al. [16]	2257	6.0	101	−0.80	(−1.54, −0.06)	Participants who walked more than 2 miles per day: 76 (3.6) Participants who walked less than 0.25 miles per day: 77.4 (4.4)	Participants who walked more than 2 miles per day: 17% n = 78 Participants who walked less than 0.25 miles per day: 19% n = 105	0%	Physical Activity Index
Yoshitake et al. [17]	828	7.0	42	−1.71	(−2.94, −0.49)	Men: 73 (5.6) Women: 74 (6.1)	n/a	60% n = 493	Categorized self-reported physical activity levels
Ravaglia et al. [18]	749	3.9	54	−0.36	(−1.11, 0.40)	73.2 (6.0)	16% n = 123	54% n = 401	Paffenbarger physical activity questionnaire

### Statistical analysis

Due to minimal between study heterogeneity, a fixed-effects model was used for this meta-analysis. Effect sizes were calculated for each outcome measure to compare the most physically active with the least active group. Effect measures, the risk ratios and hazard ratios, were log transformed to reduce skewness and all analyses include 95% confidence intervals (CI). Note that hazard ratios were combined with risk ratios. Although hazard ratios differ in that they interpret instantaneous risk, which may not be constant throughout a follow up period, they can be interpreted similarly to risk ratios under the conditions encountered in these prospective studies [19]. For both measures, a value of one implies no significant difference in risk between the physically active and physically inactive groups.

## Results

**Figure 2:**
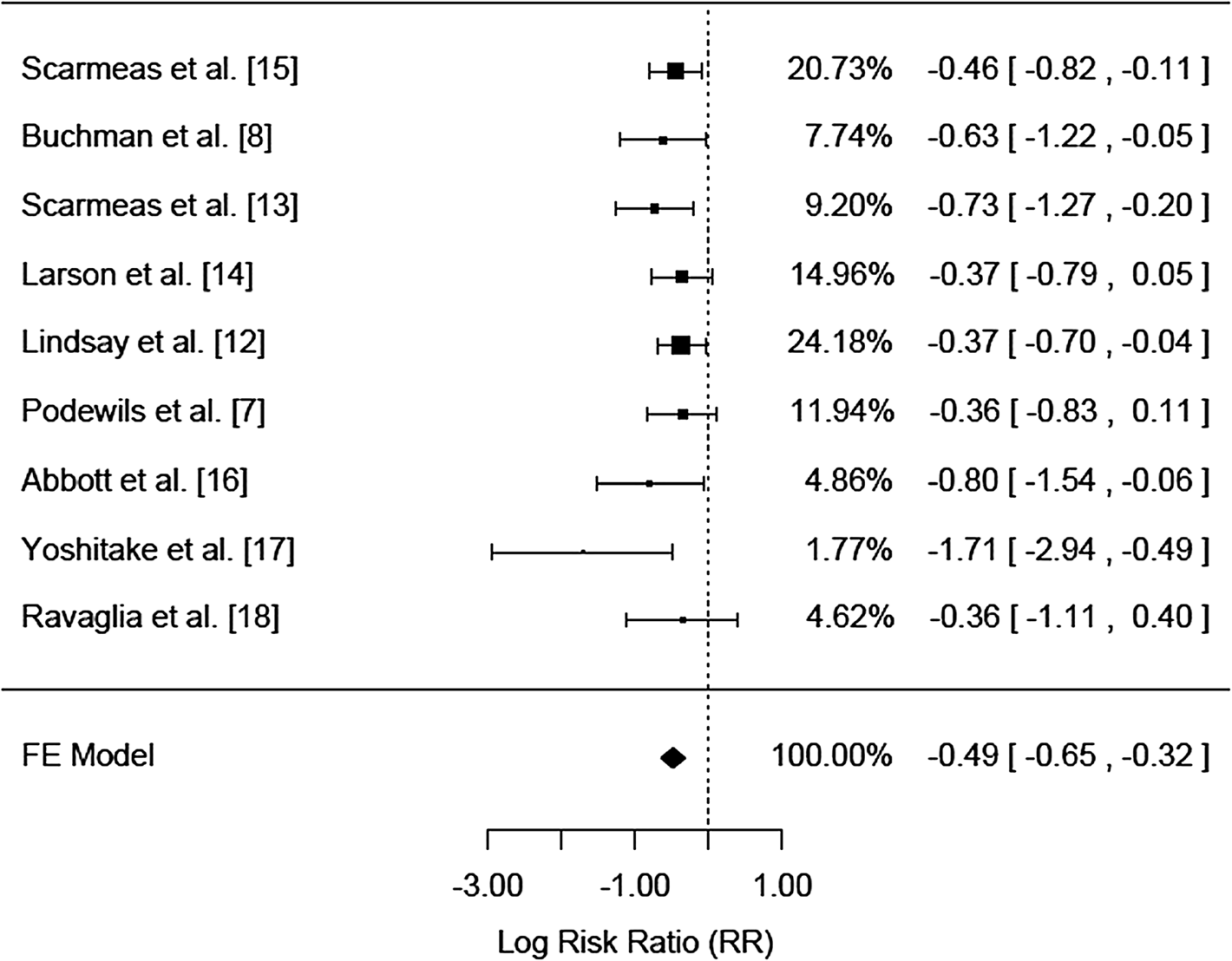
**Caption: physical activity and the reduction of risk in developing Alzheimer’s disease.**

The fixed effects log risk ratio was calculated as −0.49 (95% CI −0.65 to −0.32) which corresponds to a fixed effects risk ratio of 0.61 (95% CI 0.52-0.73) indicating a statistically significant overall reduction in risk of Alzheimer’s disease in physically active older adults compared to their non-active counterparts (Figure 2). Heterogeneity between the studies was estimated as zero, supporting the use of the fixed effects model in this meta-analysis. Furthermore, six of the nine studies included in the meta-analysis demonstrated a statistically significant reduction in risk of Alzheimer’s disease in the physically active older adults [8,12-14,16,17].

## Discussion

The results of this meta-analysis indicate that physical activity may be an important protective factor against Alzheimer’s in adults 65 years and older. Specifically, all point estimates of the log risk ratios in the included studies showed that the most physically active group was less likely to develop Alzheimer’s disease than their less active counterparts. Moreover, this association was statistically significant for six of the included nine studies.

Given the results of this meta-analysis, public health should consider placing a greater emphasis on encouraging physical activity participation in older adults. Evidence already indicates that moderate to high levels of physical activity are associated with an improved quality of life and improved impact on mood and depression in older adults, [20,21]. In addition, physical activity is effective at improving sarcopenia and reducing functional impairment associated with senescence [22]. Given that modern day pharmaceutical interventions are incapable of reversing the effects of Alzheimer’s disease, a greater shift towards prevention and delaying the onset of Alzheimer’s should be emphasized [22,23].

The exact biological mechanism by which physical activity preserves cognitive function in older adults is still unknown. Some evidence attributes physical activity with an increased blood flow to the brain, while alternative evidence suggests that regular exercise is associated with increases in molecular growth factors, such as brain-derived neurotrophic factor (BDNF) and insulin-like growth factor (IGF-1), both of which play a crucial role in neuroprotection, as well as enhancing neurotransmitter functions [12,15]. Smith et al. [24] found that individuals with the APOE protein, and therefore at high genetic risk for Alzheimer’s disease, benefited from an 18-month physical activity intervention. During the 18-month interval, regular levels of physical activity inhibited atrophy of the hippocampus, preserving hippocampal volume and protecting against impairments in episodic memory [24].

One of the strengths of this meta-analysis is the large sample size. Demographically this sample population is very similar to a real world population, so results from this meta-analysis are externally valid. RRs and HRs included in this meta-analysis were also pre-adjusted for confounding variables, reducing potential bias in the fixed effects model. A panel of physicians or neurologists, who examined available cognitive and clinical data, typically confirmed diagnosis of Alzheimer’s disease. Moreover, a definitive requirement of Alzheimer’s disease, instead of dementia more broadly, helps ensure that studies remain comparable.

One of the main limitations of this analysis was that most of the studies included relied on self-reported physical activity levels, which can be prone to recall bias, particularly given the older age of the adults included in this meta-analysis. The only exception is Buchman et al. [8] whose participants wore actigraphs for up to ten days to assess physical activity levels. Details of the physical activity assessment in each study are outlined in Table 2. Furthermore, physical inactivity may be a prodromal symptom of Alzheimer’s disease. To minimize the effects of reverse causality, most studies took this into consideration by conducting cognitive tests on their participants and including only the top scoring older adults or by excluding the results of adults who exhibited early signs of cognitive decline at follow up. However, given the relatively short follow up periods for most of the included studies, reverse causality could still have an effect. Furthermore, a universally standard questionnaire to capture physical activity level was not used between studies. Adopting the use of either the International Physical Activity Questionnaire [25] or the Global Physical Activity Questionnaire [26] in future research could potentially lessen bias in physical activity levels between studies. Future research that can uniformly capture physical activity intensities and duration over an extended time period is needed to compare its effect on cognitive decline in older adults. In addition, we adopted the simplifying assumption that risk ratios and hazard ratios were measuring the same underlying metric of risk in the included studies. As the hazard ratio consistently exceeds the relative risk when the effect measures are less than one [19], this results in a more conservative analysis that is slightly biased towards showing no effect under the rare disease assumption and small to moderate effect sizes [19].

**Table 2:** **Description of physical activity measurements in each study**

**Study**	**Assessment of Physical Activity**
Scarmeas et al. [13]	Metabolic equivalents were assigned to 3 different categories of activities: vigorous, moderate or light. Low physical activity defined as: 0 hours per week. High physical activity defined as: 1.3 hours of vigorous, 2.4 hours of moderate, 4 hours of light physical activity. Participants self- reported their activity levels.
Buchman et al. [8]	Daily physical activity assessed with Actigraphy for up to ten days. Total daily physical activity was the daily sum of all activities recorded.
Scarmeas et al. [14]	Metabolic equivalents were assigned to 3 different categories of activities: vigorous, moderate or light. Low physical activity was defined as: 0 hours per week. High physical activity was defined as: 1.3 hours of vigorous, 2.4 hours of moderate, 4 hours of light physical activity per week. Participants self- reported their activity levels.
Larson et al. [15]	Participants reported the number of days per week engaged in the following activities for at least 15 minutes: walking, hiking, bicycling, aerobics, swimming, weight training or stretching. Study was divided between those who do 3+ activities per week versus those who did fewer than 3 activities per week.
Lindsay et al. [12]	Participants asked if they engaged in regular physical activity (yes/no). Physical activity was not explicitly defined.
Podewils et al. [7]	Participants listed frequency and duration of 15 activities over the previous 2 weeks. Activities were assigned metabolic equivalents in accordance with intensity level.
Abbott et al. [16]	Participants were asked about the average distance they walked per day. The most active group walked more than 2 miles per day while the least active walked less than 0.25 miles per day.
Yoshitake et al. [17]	4 categories of physical activity for leisure and for work. The physically active group was defined as reporting exercise daily during the leisure period or participating in daily moderate to severe activity at work.
Ravaglia et al. [18]	Participants asked about city blocks walked, flights of stairs climbed and frequency/duration in various sports activities per week in the past year. Each activity was assigned a metabolic equivalent.

Finally, note that these results are consistent with a recently published meta-analysis by Norton et al. [27], who showed that physical inactivity is a significant contributor to the risk of developing Alzheimer’s disease using the population attributable risk, as well as Aarsland et al. [28] who previously showed the protective effect of physical activity on the prevention of Alzheimer’s disease. However, note that this analysis is unique as it includes only older adults (over 65 years of age) in the study population.

## Conclusions

Due to increases in life expectancy, age-related diseases are on the rise [22]. This meta-analysis has shown that physical activity is associated with a reduced risk of developing Alzheimer’s disease in adults 65 years and older. Given the limited treatment options, an emphasis on health interventions to encourage older adults to incorporate physical activity in their lives may be a viable and safe way to slow the rising rates of Alzheimer’s disease. Nonetheless, future mechanistic studies are needed to clarify the biological phenomena behind the protective effects of physical activity on the incidence of Alzheimer’s disease.

## Additional file

Additional file 1:
**PRISMA 2009 Checklist.**
